# VEXAS Syndrome in a Patient with Myeloproliferative Neoplasia

**DOI:** 10.1155/2023/6551544

**Published:** 2023-02-25

**Authors:** Janne Austestad, Tor Magne Madland, Miriam Sandnes, Torjan Magne Haslerud, Andreas Benneche, Håkon Reikvam

**Affiliations:** ^1^Department of Medicine, Haukeland University Hospital, N-5021 Bergen, Norway; ^2^Department of Rheumatology, Haukeland University Hospital, N-5021 Bergen, Norway; ^3^Department of Clinical Science, University of Bergen, N-5021 Bergen, Norway; ^4^Department of Nuclear Medicine, Haukeland University Hospital, N-5021 Bergen, Norway; ^5^Department of Medical Genetics, University of Bergen, N-5021 Bergen, Norway

## Abstract

VEXAS syndrome stands for vacuoles, E1 enzyme, *X*-linked, autoinflammatory, somatic syndrome. The syndrome is a combined hematological and rheumatological condition caused by a somatic mutation in the *UBA1*. There is an association between VEXAS and hematological conditions such as myelodysplastic syndrome (MDS), monoclonal gammopathies of uncertain conditions (MGUS), multiple myeloma (MM), and monoclonal B-cell lymphoproliferative conditions. There are not many descriptions of patients having VEXAS in combination with myeloproliferative neoplasm (MPN). With this article, we want to present a case history of a man in his sixties with a *JAK2*V617F mutated essential thrombocythemia (ET) developing VEXAS syndrome. The inflammatory symptoms occurred three and a half years after the ET diagnosis. He started to experience symptoms of autoinflammation and an overall worsening of his health, and blood work showed high inflammatory markers, leading to repeated hospitalizations. His major complaint was stiffness and pain, and high dosages of prednisolone were necessary to obtain pain relief. He subsequently developed anemia and significantly variable levels of thrombocytes, which previously were at a steady level. To evaluate his ET, we made a bone marrow smear demonstrating vacuolated myeloid and erythroid cells. Having VEXAS syndrome in mind, genetic testing identifying the *UBA1* gene mutation was performed, thus confirming our suspicion. The work-up with myeloid panel on his bone marrow identified genetic mutation in the *DNMT3* too. After developing VEXAS syndrome, he experienced thromboembolic events with both cerebral infarction and pulmonary embolism. Thromboembolic events are also common in *JAK2* mutated patients, but in his case, they presented first after VEXAS had developed. Throughout the course of his condition, several attempts with prednisolone tapering and steroid sparing drugs were tried. He could not get pain relief unless the combination of medications included a relatively high dose of prednisolone. Currently, the patient uses prednisolone, anagrelide, and ruxolitinib, with partial remission and fewer hospitalizations and more stabilized hemoglobin and thrombocytes.

## 1. Introduction

VEXAS syndrome is a recently described disease entity caused by somatic mutation in the *UBA1* coding gene, localized on the X-chromosome. Patients carrying this mutation present heterogeneous disease spectrum, encompassing both hematological and rheumatic manifestations. The disease is associated with high morbidity and mortality rates. Most described patients have coexisting hematological diseases such as myelodysplastic syndrome (MDS), monoclonal gammopathies of uncertain significance (MGUS), multiple myeloma (MM), or other monoclonal B-cell lymphoproliferative conditions. There are only a few descriptions of coexistence of myeloproliferative neoplasms (MPNs) in VEXAS syndrome in the literature. We present a case of a male with essential thrombocythemia (ET) that develops symptoms leading to the diagnosis of coexisting VEXAS syndrome [1].

## 2. Case Presentation

**Figure 1:**
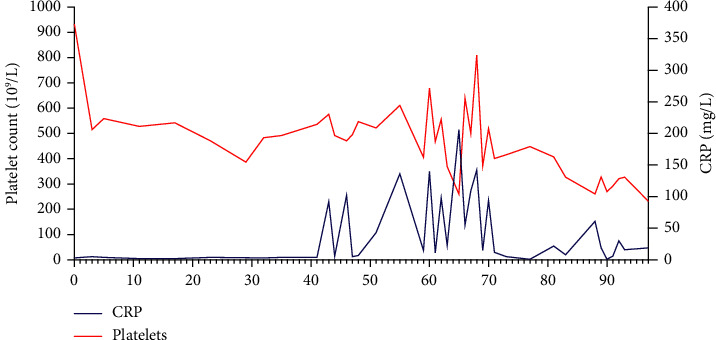
Thrombocyte levels (red line) and C-reactive protein (CRP) (blue line) during the disease course. It also illustrates the uncomplicated levels during the first 40 months, progressing into spiking levels as the clinical course changes and turns more complicated.

Eight years ago, a man in his sixties got the diagnosis of ET, after a diagnostic workup due to persistent thrombocythemia. Blood count at diagnosis revealed hemoglobin (Hb) 15.0 g/dL (reference value 13.4–17.0), leukocytes 7.1 × 10^9^/L (4.1–9.8), and thrombocytes 932 × 10^9^/L (145–348). Tests identified *JAK2*V617F mutation, and a bone marrow biopsy demonstrated classical morphological signs of ET. After standard risk classification, initiation of treatment with acetylsalicylic acid and hydroxyurea (HU) followed. During the first years, there were no thromboembolic events and only minor complications related to HU-induced bone marrow depression with leukopenia. As illustrated in Figure 1, there were steady thrombocyte levels for the first four years. At routine checkups, he reported a good quality of life without noteworthy side effects.

**Figure 2:**
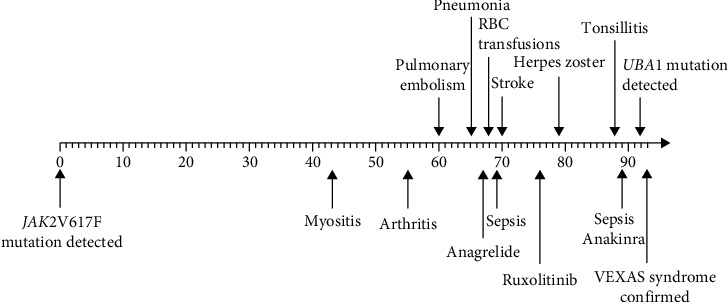
Timeline in months showing the most significant events of his disease progress from time of the ET diagnoses until the complicated course of VEXAS syndrome. RBC: red blood cells.

Four years after the initial diagnosis of ET, the patient's symptomatology was suddenly evolving, resulting in several hospitalizations and further diagnostic work. During the first disease attack, the reason for hospitalization was fever and left-sided abdominal pain. The suspicion of the urinary tract infection arose from clinical and laboratory findings, and antibiotics were the choice of treatment. Although the infection was improving, he did not fully recover. He experienced increasing pain and weakness in both lower extremities, and he gradually developed global stiffness. A magnetic resonance imaging (MRI) scan was performed, revealing edematous changes of the muscles compatible with the diagnoses of moderate to severe myositis. The working diagnosis of atypical myositis related to infection was the background for initiating prednisolone 20 mg, upon which he showed a clinical response. After therapeutic dosages, a careful tapering followed, but immediately below the dose of 20 mg, his clinical conditions deteriorated. He could not tolerate lower dosages. The following year, he had a traumatic metacarpal fracture of his left hand, which was complicated with infections and recurrent abscesses. At the same time, he developed night sweats, weight loss of eight kg, bunions under his skin on his forearms, and an additional migrating maculopapular rash. Simultaneously, the pain in his lower extremities reoccurred, now with the involvement of the ankles. There was a thorough examination done, looking for differential diagnoses including various infectious, inflammatory, and malignant conditions without diagnostic findings. Typical myositis, atypical polymyalgia rheumatic, or paraneoplastic inflammatory conditions were the native diagnoses that clinicians considered. The situation was further complicated with a pulmonary embolism that was treated with warfarin (Figure 2).

Again, to assure proper rheumatological diagnostic work-up, taper prednisolone. This, however, led to recurrent intolerable stiffness and pain. The symptoms evolved to include shoulders, elbows, and wrists and was somewhat accelerated. The increase in inflammatory markers was significant, and he could not tolerate a prednisolone dose below 20 mg. Figure 1 shows the steadiness of the thrombocytes following diagnoses of ET, until the inflammatory process started, as demonstrated with the spiking course of inflammatory markers, i.e., CRP, the thrombocytosis was also highly variable and difficult to manage.

**Figure 3:**
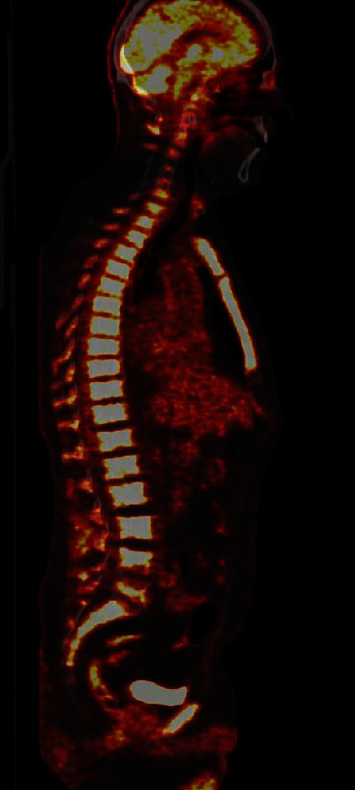
[F^18^]FDG-PET-CT showing intense uptake in the central bone marrow.

In an attempt to identify the underlying inflammatory condition, the prednisolone dosage was 5 mg. The severity of his condition evolved during the tapering. He became febrile and generally ill, and sepsis was suspected at admission. Hb levels decreased, and he became dependent on erythrocyte transfusions, and leukopenia and variable thrombocyte levels accompanied. [F^18^]Fluoro-deoxy-glucose-positron emission tomography/computed tomography ([F^18^]FDG-PET/CT) revealed no signs of vasculitis or other inflammatory disease, although it demonstrated generalized diffuse increased FDG-uptake in the bone marrow (Figure 3) and diffuse increased metabolic activity/FDG-uptake in the spleen. About 60 months after the initial diagnosis of ET, a bone marrow biopsy showed no signs of transformation to secondary myelofibrosis (MF) or acute myeloid leukemia (AML). 10 months following the pulmonary embolism, he suffered yet another thromboembolic event in the form of cerebral apoplexy (Figure 2). The patinent's main clinical and laboratory findings are present in Table 1.

As ET seemed to evolve, there was an attempt to add anagrelide to lower platelet counts and later ruxolitinib for utilizing of the anti-inflammatory trait. He was showing signs of Cushing's syndrome, underlining the complicated course of long-term usage of prednisolone. Introduction of methotrexate as a steroid sparing alternative came about. However, he still could not tolerate a tapering of prednisolone that led to the termination of methotrexate due to a lack of desired effect. After several hospitalizations with diffuse tonsillitis, the rheumatologist added anakinra to his preexisting treatment regimen, now including prednisolone, anagrelide, and ruxolitinib. This combination accordingly stabilized the clinical course of the disease as well as the hematological values.

**Figure 4:**
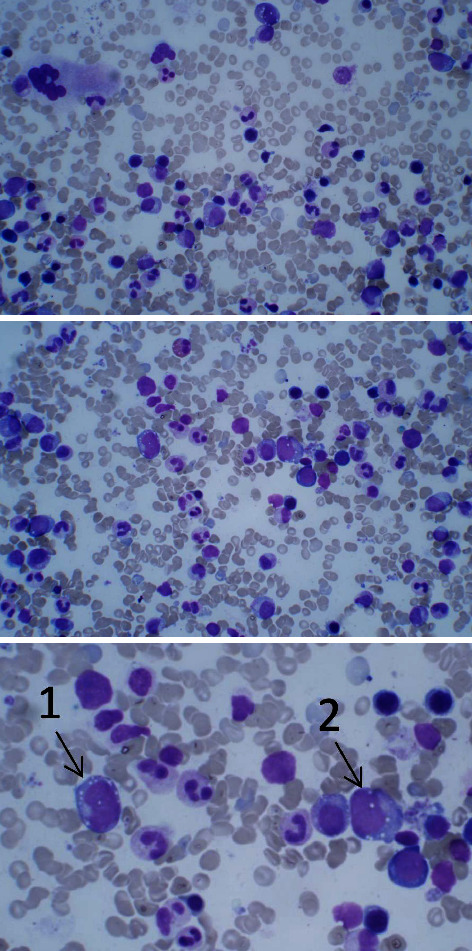
Picture from bone marrow smear demonstrating vacuolization in erythroid and myeloid cells, next to a normal megakaryocyte.

**Figure 5:**
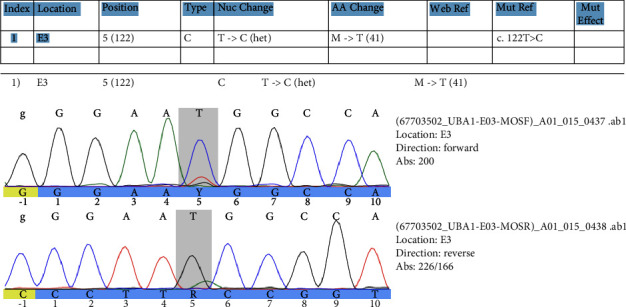
DNA sequencing report illustrating the mutation in the fifth position. The variant p.Met41Thr exists as the dominant allele.

Based on the patient's age, gender, clinical picture, and hematological disease, suspicion of the recently described VEXAS syndrome arose. A new bone marrow smear was performed demonstrating vacuolization of granulocyte- and erythroid-precursors (Figure 4). Myeloid panel of bone marrow cells furthermore demonstrated a *DNMT3A* variant, and the previously described *JAK2*V617F mutation. Sequencing of *UBA1* confirmed mosaicism for the known pathogenic missense variant p.Met41Thr in *UBA1* (Figure 5), thus confirming VEXAS syndrome. The current medication to meet his VEXAS syndrome is prednisolone varying between 15 and 20 mg/day, anagrelide 1.5 mg/day, and ruxolitinib 10 mg/day. He reports a good quality of life, but with intermittent pain. The thrombocyte levels are stable, and his leukopenia is currently not clinically significant. We revised allogenic stem cell transplantation as a possible curative therapy and concluded that the patient is not alleged.

## 3. Discussion

VEXAS stands for vacuoles, E1 enzyme, X-linked, autoinflammatory, somatic syndrome. Typically, the onset of the inflammatory syndrome occurs in men over the age of 60. The characteristics of VEXAS syndrome are a refractory inflammatory condition combined with cytopenias and morphological vacuolization of the myeloid and erythroid precursor cells. Majority of the patients are men, but there are also cases described in elderly women with monosomy *X*, compatible with the location of the mutated gene on the *X* chromosome [2, 3]. In a VEXAS syndrome cohort, the dominant disease-causing variant was found to be the p.Met41Thr variant of the *UBA1* gene [4]. Mutant myeloid cells are believed to drive the inflammation in patients with VEXAS syndrome, as analyses have revealed activated inflammatory pathways consistent with myeloid-induced inflammation, including tumor necrosis factor (TNF), interleukin-6 (IL-6), and interferon-gamma (INF-gamma). The p.Met41 variant causes decreased ubiquitination and increased inflammation due to the production of an inactive isoform. The mutations in the hematoprogenitor cell population are restricted to maturing myeloid cells [5].

VEXAS syndrome may present with fever, pneumonia, chondritis, arteritis, and neutrophilic dermatitis. Patients often meet diagnostic criteria for rheumatological diseases such as Sweet syndrome, relapsing polychondritis, adult-onset Still's disease, giant cell arteritis, and polyarthritis nodosa [2, 5, 6]. Initially, our patient presented with inflammatory symptoms such as weakness, stiffness, and pain. Furthermore, demonstration of edema correlating to myositis could be identified through imaging techniques, making myositis the initial presentation of an inflammatory disease. Throughout the course, he developed a migrating maculopapular rash. The symptoms were clearly a result of an inflammatory process, as inflammatory markers increased simultaneously with his symptoms. In Figure 1, we demonstrate the steady levels of thrombocytes and CRP after the ET diagnosis was set, and approximately 40 months later, the levels are spiking as symptoms of inflammation arise. Thromboembolic events are common in *JAK2*-mutated MPN and are shown to also be common in VEXAS syndrome [7]. Our patient had his first known thromboembolic event, as inflammation was established, leaving the time of ET uncomplicated.

The *JAK2* mutation driving the MPN preceded the occurrence of the symptoms suggestive of VEXAS *UBA1* mutation. The findings in myeloid panel genetic analyses demonstrated a *DNMT3A* mutation. Hematopoietic stem cells clonal expansion most commonly involves epigenetic regulator genes such as *DNMT3A*, *TET2*, and *ASXL1*. These mutations select the hematopoietic stem cells for clonal expansion. Healthy individuals can carry these mutations without presenting disease, defined as clonal hematopoiesis of intermediate potential (CHIP). In one study, *DNMT3A* mutation was seen in 47.4% with VEXAS syndrome: *TET2* in 20.0% and *ASXL2* in 13.3% [5]. Driver mutations such as *JAK2* are estimated to occur early in life, including in utero period. The mean latency between *JAK2* acquisition and diagnosis was 30 years in Williams et al. article, suggesting that early driver mutation underlie adult MPN [8].

Recent research indicates that the *JAK2*V617F mutated hematopoietic clones occur very early in life, even in utero, and can be detected before the development of overt MPN [8]. Acquisition of the *JAK2*V617F mutation also predisposes to the development of additional mutations and sister clones that are drivers of myeloid neoplasms and potential transformation [8]. For the development of VEXAS syndrome and acquisition of the *UBA1* mutation in patients with *JAK2*V617F mutation or other driver mutations, it is currently unknown when the time course of these events occurs in relation to the development of fulminant phenotypic disease. This patient was administered HU to manage thrombocytosis. Based on the available evidence, antineoplastic agents such as this drug may induce tumors even when used in therapeutic dosages, as they are cytotoxic and genotoxic [9]. To our knowledge, there are only a few patients with MPN and concomitant VEXAS syndrome, and both *CALR* and *JAK2* mutations are now described [1]. We can speculate that the administration of HU has significance to the second gene mutation, although there is no supporting evidence for this correlation.

The inflammation ceased with moderate doses of prednisolone and reoccurred rapidly with tapering. Our patient clearly demonstrated a need for a dosage of prednisolone of 20 mg, and methotrexate did not induce a steroid sparing effect. Steals et al. describe a case with similar findings of prednisolone dependency and no effect of methotrexate, as a steroid sparing drug [6]. This is also in accordance with VEXAS syndrome being refractory to most anti-inflammatory approaches [10–12]. There is a clinical heterogeneity within the VEXAS patient population, and combined with the limited experience, treatment recommendations and prognostic markers are still lacking. The only potentially curative treatment is allogeneic stem cell transplantation; nevertheless, this is often not feasible due to advanced age, which is associated with a high risk of treatment-related morbidity and mortality in the transplantation setting. Another strategy is to target the inflammatory cascade by inhibition of proinflammatory cytokines, such as TNF, IL1, and IL6, or by targeting effector cells through JAK inhibitors [13]. In our patients' case, who was not a candidate for allogenic stem cell transplantation, strategies with combined medical treatment were followed in order to manage symptoms [14].

Concurrently with the inflammation, dysplasia, and vacuoles in the myeloid and erythroid precursor cells in a dysplastic bone marrow were demonstrated (Figure 4). VEXAS syndrome is most often manifested concomitant to premalignant or malignant hematological conditions, and MDS seems to predominate [15]. In contrast, the diagnosis of our patient was classical MPN, and the ET diagnosis was confirmed by classical morphological signs and persistence of the *JAK2*V617F mutation. Obiorah et al. describe 16 cases of VEXAS syndrome, upon which ten patients had low-risk MDS, two had MM, two had MGUS, and two had other monoclonal B-cell neoplasms. As many as ten of these 16 patients had experienced thromboembolism [2, 11, 15]. Additionally, in a single-center cohort of 147 patients with vasculitis, the diagnosis of VEXAS syndrome was confirmed in only three patients who met the definite diagnostic criteria of MDS with normal karyotype [16].

## 4. Conclusion

Our case report describes a man with known *JAK2*-mutated ET who has steady levels of thrombocytes. His condition is maintained successfully with hydroxyurea for a few years, and he has a good quality of life. There is somewhat of a sudden change in his health condition with inflammation-related pain thromboembolic events and migrating skin bunions. He needs a moderate dosage of prednisolone to manage the pain associated with inflammation. There are several attempts to introduce steroid sparing drugs, although so far not successful. The combination of prednisolone, anagrelide, and ruxolitinib is his current treatment regimen, and it has stabilized his condition. Prednisolone to manage the inflammation, anagrelide for the thrombocytosis, and ruxolitinib has somewhat of an anti-inflammatory effect that is beneficial. The treatment option allogenic stem cell transplantation is a possible curative treatment for this group. This patient is considered unsuited for this treatment approach.

## Figures and Tables

**Table 1:** Organ manifestations of VEXAS syndrome in the present patient.

Organ systems	Manifestations
Skin	Migrating exanthem
	Maculopapular rash

Musculoskeletal	Stiffness and pain
	Edematous changes shown on CT scan

Circulation	Cerebral infarction
	Pulmonary embolism

Bone marrow	Changes correlating to ET
	Vacuolization of myeloid and erythroid cells

Immunological	Fever and night sweats
	Increase in CRP
